# Monitoring Respiratory Motion during VMAT Treatment Delivery Using Ultra-Wideband Radar

**DOI:** 10.3390/s22062287

**Published:** 2022-03-16

**Authors:** Anwar Fallatah, Miodrag Bolic, Miller MacPherson, Daniel J. La Russa

**Affiliations:** 1 School of Electrical Engineering and Computer Science, University of Ottawa, Ottawa, ON K1N 6N5, Canada; afallata@uottawa.ca; 2 Department of Radiology, Division of Medical Physics, Faculty of Medicine, University of Ottawa, 501 Smyth Road, Box 232, Ottawa, ON K1H 8L6, Canada; mmacpherson@toh.ca; 3 The Ottawa Hospital Research Institute, 501 Smyth Road, Box 511, Ottawa, ON K1H 8L6, Canada; 4 Radiation Medicine Program, The Ottawa Hospital, 501 Smyth Road, Box 927, Ottawa, ON K1H 8L6, Canada; dlarussa@toh.ca; 5 Department of Physics, Carleton University, 1125 Colonel By Drive, Ottawa, ON K1S 5B6, Canada

**Keywords:** UWB radar, removing interference, breathing pattern classification

## Abstract

The goal of this paper is to evaluate the potential of a low-cost, ultra-wideband radar system for detecting and monitoring respiratory motion during radiation therapy treatment delivery. Radar signals from breathing motion patterns simulated using a respiratory motion phantom were captured during volumetric modulated arc therapy (VMAT) delivery. Gantry motion causes strong interference affecting the quality of the extracted respiration motion signal. We developed an artificial neural network (ANN) model for recovering the breathing motion patterns. Next, automated classification into four classes of breathing amplitudes is performed, including no breathing, breath hold, free breathing and deep inspiration. Breathing motion patterns extracted from the radar signal are in excellent agreement with the reference data recorded by the respiratory motion phantom. The classification accuracy of simulated deep inspiration breath hold breathing was 94% under the worst case interference from gantry motion and linac operation. Ultra-wideband radar systems can achieve accurate breathing rate estimation in real-time during dynamic radiation delivery. This technology serves as a viable alternative to motion detection and respiratory gating systems based on surface detection, and is well-suited to dynamic radiation treatment techniques. Novelties of this work include detection of the breathing signal using radar during strong interference from simultaneous gantry motion, and using ANN to perform adaptive signal processing to recover breathing signal from large interference signals in real time.

## 1. Introduction

In external beam radiotherapy, particularly of the thorax and abdomen, the monitoring or mitigation of patient breathing motion is often an integral part of an accurate treatment delivery process [1,2]. Using respiratory gating and breath hold techniques during lung radiotherapy, for instance, can significantly reduce pulmonary, cardiac and esophageal late toxicities [3,4]. Similarly, for patients with left-sided breast cancer, proper motion management during whole-breast radiotherapy enables safe implementations of deep-inspiration breath hold (DIBH) treatment techniques, subsequently reducing the risk of treatment-related cardiac toxicity [5].

Given the numerous indications for respiratory motion management and possibly gating, it is no surprise there exists several commercial solutions available for breathing motion management [6]. Despite their wide adoption, many of these technologies rely on patient-worn accessories [7] or high-cost peripheral devices [8]. Further, from the experience of the authors working in the clinical setting, all solutions introduce a risk of random failure, while some also place restrictions on clinical setups owing to their mode of operation or the equipment involved. In principle, eliminating the need to use patient-worn tracking accessories will contribute to minimizing patient appointment times, and allow for more treatments to benefit from breathing motion tracking.

In this work, we explore the use of a low-cost, ultra-wideband (UWB) radar system for monitoring breathing motion in the radiotherapy setting. Radar devices are already being applied as a non-invasive, contactless solution for vital signs monitoring. Non-invasive monitoring of vital physiological activities such as breathing and heartbeat [9,10], and human physical activity [11] have been accomplished using UWB and continuous-wave (CW) radars. The ability to detect respiration-related motion in a human body has been reported in many works for UWB radar [12,13,14] and for CW radar [15]. Due to their low cost and high reliability, radar systems also have the potential to serve as an accurate monitor of breathing motion during radiotherapy. A detailed overview of the use of radar in radiotherapy is given by Li et al. [16]; however, most of the implementations are based on continuous-wave radar due to its configurability [17,18,19,20,21,22,23]. More recently, Masashi et al. reported on the simulation of UWB radar system for distinguishing between costal and abdominal breathing [20,24]. Among the radar systems evaluated, multi-radar systems are preferred due to their increase in positional accuracy using beamforming [19,20,21,24,25,26]. Classification of human activities and breathing patterns in presence of interference was addressed for monitoring people in smart homes or in prisons [27]. In comparison with works that deal with motion of both radar and the target [28], this work is limited to stationary radar as well as stationary target but the quality of the signal is affected by the external interference.

Recently, there have been several works that perform respiratory pattern classification based on radar signals. Abnormal breathing patterns are commonly classified including Biot’s respiration, Cheyne Stokes respiration, Kussmaul breathing and apneas [26]. Classifiers that were used include support vector machines, random forests and other classifiers [29,30]. In this paper, the classes are specifically targeted for respiration gating during the radiation treatment and they include no breathing, breath hold, free breathing and deep inspiration classes.

In prior investigations of using radar in the radiotherapy setting, the distance between the radar and the target to be positionally tracked was minimized in order to increase detection accuracy and avoid interference from the nearby treatment machine. Distances less than one meter were typical, which is acceptable is easily accommodated in the majority of clinical setups [31,32,33,34,35]. Performance is further improved with hardware selection or development. In some examples, a Fermi antenna was used to implement multi-array systems in a compact size [20]. Others employed a DC-coupled method to achieve a stationary output and accurate performance [22,23].

In this work, we explore the use of a single, low-cost UWB radar for monitoring breathing motion in the radiotherapy setting, with the goal to isolate breathing motion from the large interference background due to linac motion and operation. In addition, we aim to develop signal processing algorithms that 1. remove interference from the radar signal due to gantry motion and linac operation using machine learning approaches, and 2. accurately classify different breathing patterns. In order to make the system commercially viable and generally accessible, off-the-shelf inexpensive (<$400) radar is used. This is different from other approaches presented in the literature that do not consider gantry movement, rely on radars with multiple antennas, or use custom radar systems.

All data presented in this manuscript is experimental data. The term “simulate” or “simulation” is used to refer to the respiratory motion phantom that was used to mimic breathing motion in a manner akin to a real patient. The data collected during the study and the code in Matlab (The MathWorks Inc., Natick, MA, USA) will be posted online to allow for and to support the reproducible research.

## 2. Materials and Methods

### 2.1. Experimental Design

In the present experiment, we simulate breathing motion during delivery of a VMAT treatment using a respiratory motion phantom (‘RMP’; Quasar, Modus Medical, London, ON, Canada) [36]. We utilized a representative VMAT treatment delivery to introduce noise in the radar-measured signal. The delivery was cloned from the treatment of a real patient receiving radiotherapy for prostate cancer. This particular treatment involved the delivery of 200 cGy over 360 degrees rotation, requiring the linac gantry and multi-leaf collimators (MLC) to move quickly during treatment, and thus producing the largest possible noise signal one would expect to encounter in these treatment conditions. The gantry speed varies throughout the rotation, and the MLC leaf patterns that collimate the beam are unique (and patient-specific) at each gantry angle.

A single UWB radar, which is a low-energy and short-range technology, is used to measure the combined signal from the RMP and linac, where the distance between the radar and the RMP is approximately 70 cm. The radar used in this experiment is the Xethru X4M03 development kit manufactured by Novelda (Oslo, Norway). This radar uses a UWB transceiver operating in the range of 5.9–10.3 GHz and a patch antenna with 65 degree aperture in both azimuth and elevation axes. This particular radar is chosen because of its low cost, small size and high spatial resolution. Measurements of the isolated phantom motion, the isolated gantry motion during treatment delivery, and the combined motion were obtained at the radar sampling rate of 17 Hz. At each time instant, the radar receives a vector of data containing a projection of movements at different distances called range bins between the radar and the maximum distance of 2 m. Each range bin is associated with a specific distance from the radar. A raw vector of range bins across time is called a fast-slow time matrix.

**Figure 1:**
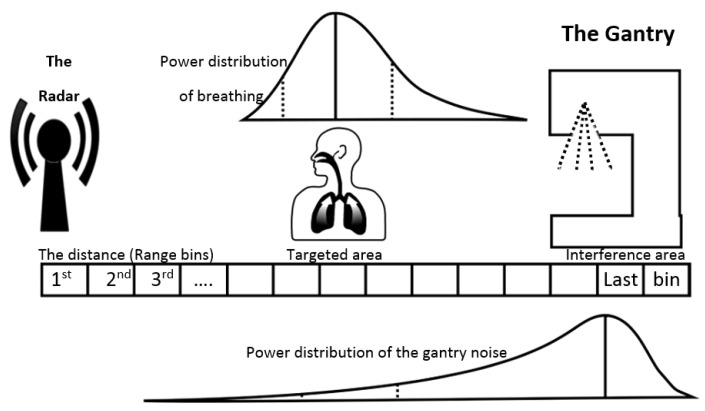
Schematic illustration of the experimental setup. The radar system was placed at the foot-end of the treatment couch facing the RMP and linac gantry. The power distributions of the RMP and gantry are broad and attributed to the relatively non-local nature of their motion.

An illustration of the experimental setup in shown in Figure 1, which also shows the power distributions of the target signal from the RMP, and interference signal from the linac. The power distribution of the breathing signal is shown only conceptually—however, the expected range is between 0 and 30 breaths per minute with the mode around 15 breaths per minute. The gantry noise occupies much larger frequency band. The range bin of the radar is small (around 5 cm) so that the breathing signal appears in multiple range bins. The same applies to the interference signals from the linac motion/operation. The magnitude of power of the radar signal from the linac can be several times larger than the magnitude of power produced by simulated respiratory motion. This low signal to noise ratio makes it very challenging to extract the signal from respiratory motion when the gantry is moving. Furthermore, the signals from the respective sources are broad, which is attributed to the fact that the source of the signals are not locally confined (i.e., they come from the RMP and gantry motion/linac operation).

Two breathing patterns were simulated with the RMP: a basic sinusoidal pattern with a frequency of 0.25 Hz and amplitude of 4 mm, and a DIBH sequence that was included with the phantom software.

### 2.2. Breathing Signal Recovery

**Figure 2:**
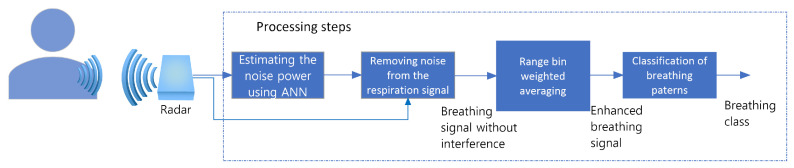
Processing stages including breathing signal recovery (first 2 processing blocks), signal enhancement block and classification of breathing patterns.

In this section, we will consider interference from the gantry as a noise contaminating desired breathing signal. The process for obtaining the breathing signal from the high noise background is divided into two main steps: (1) estimating the noise power using an artificial neural network (ANN) followed by (2) increasing the signal-to-noise (SNR) of the targeted signal by range-bins weighted averaging (RBWA). These steps are followed by classification step as shown in Figure 2. The Step 1 is implemented using two approaches that depend on the number of bins that we use for estimating breathing, referred to here as single-bin ANN, and range-bins ANN.

#### 2.2.1. Single-Bin Artificial Neural Network Noise Estimation

**Figure 3:**
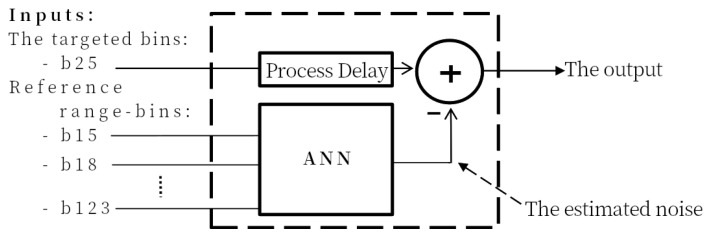
A block diagram for the SB-ANN interference (noise) estimation process.

In the first of two noise estimation methods, an ANN is used to estimate the noise from linac motion/operation in a single target range bin that has the strongest breathing power (in this case, bin 25, notated as b25). We refer to this noise estimation technique as the single-bin ANN, or SB-ANN. Seven different non-target range bins are selected at random to be the noise reference to provide a variety of samples to train the ANN. Figure 3 shows the architecture of SB-ANN system. We know in advance based on the placement of the radar which range bins correspond to breathing signal and noise and which to the noise only. In Figure 1, range bin b25 is used as a signal range bin while b15, b18 and b123 are three out of seven range bins used as noise inputs.

The network used in this work is Multi-Layers Perceptron ANN structure that has 14 inputs (7 range bins real and imaginary components), a first hidden layer of 56 neurons, and a second hidden layer of 28 neurons and two outputs. The neural network is trained to estimate the noise amplitude and phase distortion in the targeted range-bin. 70% of the samples are used for training, 15% for the validation. The achieved performance of Mean-Square-Error (MSE) = 3.17 × 10${}^{-8}$.

#### 2.2.2. Range-Bin Artificial Neural Network Signal Noise Estimation

**Figure 4:**
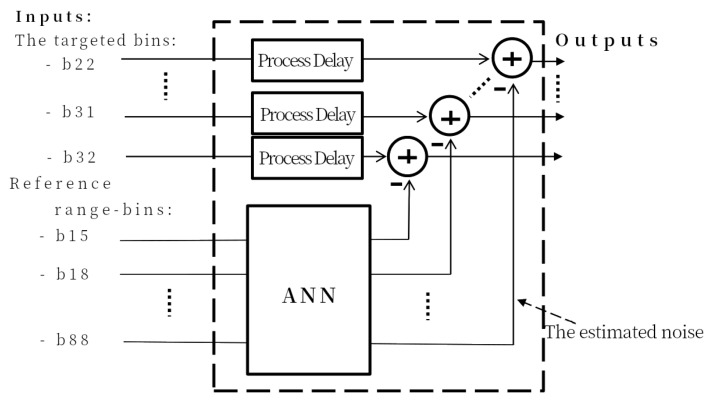
The block diagram for the RB-ANN interference (noise) estimator.

The second noise estimation process is a variation of the first. Instead of estimating the noise in only one target range bin, noise is estimated in multiple range bins to average them later. We refer to this approach as range-bin ANN, or RB-ANN. As in the SB-ANN approach described above, the RB-ANN is trained on the same noise-reference range bins to estimate the entire noise under the targeted area. Figure 4 shows the block diagram of the RB-ANN. In this case the noise is estimated in 11 channels.

The structure of the RB-ANN is the same as the SB-ANN, but now the first hidden layer has 126 neurons, the second hidden layer 22 neurons and 22 outputs. The neural network is trained to estimate the noise amplitude and phase distortion in the targeted range-bin. 70% of the samples are used for the training, 15% for the validation. The achieved performance of Mean-Square-Error (MSE) = 8.37 × 10${}^{-8}$.

#### 2.2.3. Signal Enhancement with Range Bin Weighted Averaging

Theoretically, coherent averaging of a set of signals increases the SNR in proportion to the square root of the number of trials. This fact can be exploited to enhance the target breathing signal by appropriately averaging the target range bins [37]. Since the signals from the target range bins have different powers (Figure 1), they have to be weighted before averaging. It is also possible that the signal in some range bins are phase shifted by 180${}^{\circ }$ and therefore they appear to be inverted. As such, they cannot be used for averaging unless they are inverted back. The polarity inverted signal weights were obtained using the method described by Zyweck and Bogner [37].

### 2.3. Combining ANN Noise Estimation, Range Bin Weighted Averaging and Filtering

The ANN noise estimation and range bin weighted averaging methods described in Section 2.2 can be combined to form a unified workflow for breathing signal recovery from radar measurements. The workflow starts by establishing an appropriate noise profile for the current radar location in the treatment suite. Prior to a patient’s treatment (i.e., no patient in the room), the treatment delivery is executed with the radar setup for patient breathing monitoring, and the resulting radar signals are used to select the interference range bins, train the ANN noise estimation model, and store the model and model weights in the radar profile associated with its current location. Later, with the patient set up for treatment, the target signal from patient breathing is localized in the absence of background motion (i.e., prior to treatment). From this, the appropriate target range bins containing the breathing signal are selected, as are the corresponding weights for range bin weighted averaging (Section 2.2.3). This target range bin selection and weight determination combine with the noise profile to form a breathing rate detection profile. The final step to the signal processing, involves filtering the weighted-average enhanced signal to remove residual noise using a moving average filtering. Finally, the recovered breathing pattern is classified as described in the next section.

### 2.4. Classification

The goal of classification is to reliably detect changes in breathing pattern. This is possible only after the breathing signal is filtered out so that it is clean enough to be classified. Classification is based on the k-means algorithm for finding optimal thresholds. The measured breathing patterns are divided into two main groups: with and without linac motion. These groups are then further subdivided into four subclasses based on signal power representing relative breathing amplitude, including: 1. no breathing (zero amplitude); 2. breath hold (small amplitude); 3. regular (free) breathing; and 4. deep inspiration/exhalation (large amplitude). Subclass 1 represents the situation where there is no subject in front of the radar. Subclass 2 represents the hold breathing event in which case the displacement of the chest is minimal but can still be detected with the radar. Deep inspiration and regular breathing subclasses are as described in [1].

## 3. Results

### 3.1. Uwb Radar Signals from Target Range Bins

**Figure 5:**
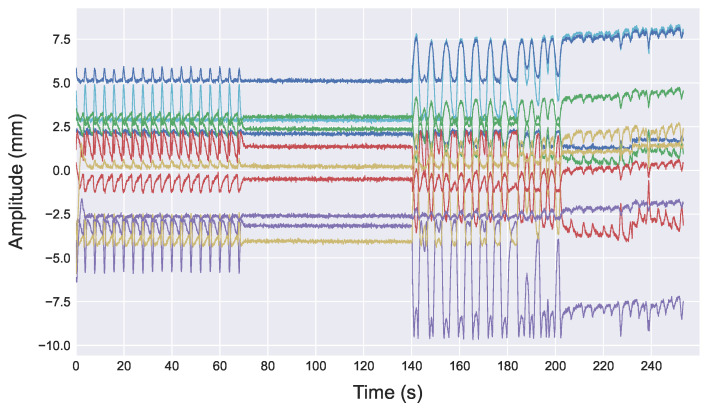
Raw radar signals from 11 consecutive range bins (distance from radar) covering an area in the vicinity of the target breathing signal from the RMP. Each line represents the signal from one of the 11 range bins.

Figure 5 shows an example of the raw radar signal (amplitude vs. time) from 11 consecutive range bins associated with the target RMP location (Figure 1) when only the RMP is moving (no simultaneous linac motion/operation). The first 70 s corresponds to a sinusoidal phantom motion, followed by another 70 s period with no motion, and then another 112 s phantom simulation of DIBH breathing pattern. The signals from each range bin have different powers, shapes, and polarity, but all have the same breathing rate.

### 3.2. Breathing Signal Recovery from High Noise Background

#### 3.2.1. Performance of the Single-Bin Artificial Neural Network Noise Estimation

**Figure 6:**
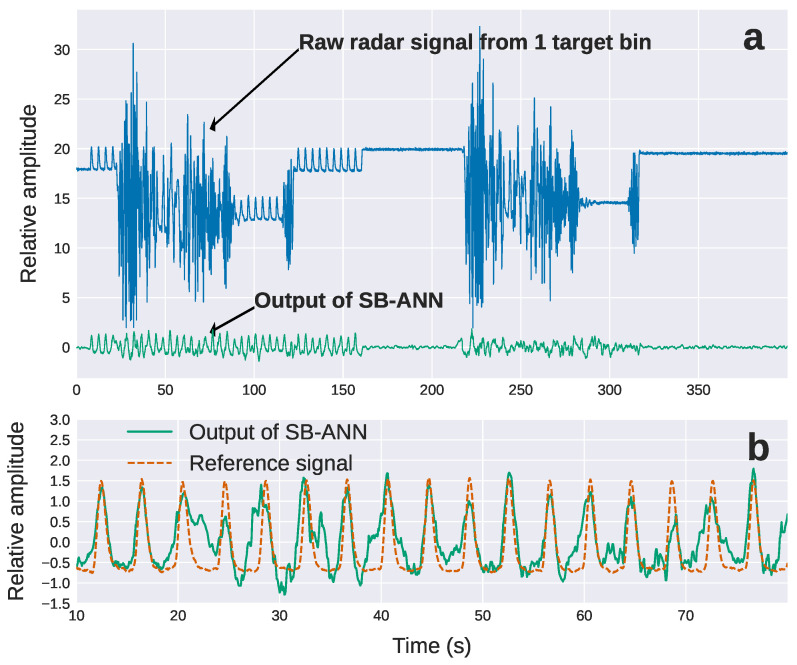
(**a**) Raw radar signal at bin 25 (blue line) and processed signal after applying SB-ANN (green line). (**b**) Processed signal (green line) from figure (**a**) in the interval between 10 and 75 s that includes gantry movement as well as the original RMP signal.

An example of the radar signal from a single target bin is shown in Figure 6 (blue line). The top figure (Figure 6a) shows the raw, unprocessed signal associated with the RMP simulating a sinusoidal breathing pattern (0 to 150 s), and the same breathing patterning with linac motion (20 to 75 s). The signal from only the linac gantry motion during the same VMAT delivery is also included (180 to 270 s).

Shown also in Figure 6a is the result of applying the SB-ANN to the raw input signal (green line). This extracted breathing pattern resembles closely the input signal from the RMP apart from residual noise between 20 and 35 s. Figure 6b shows the extracted breathing signal between 10 and 75 s, which highlights this residual noise.

#### 3.2.2. Performance of the Range-Bin Artificial Neural Network Noise Estimation

**Figure 7:**
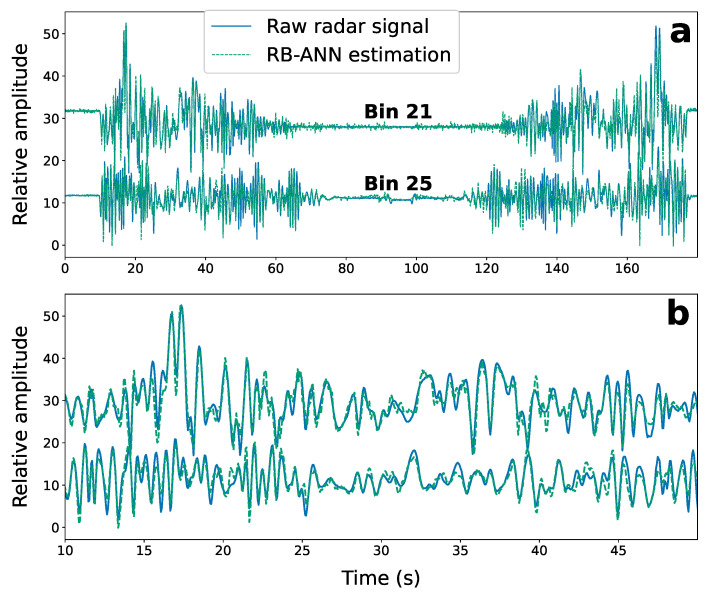
RB-ANN estimated noise (dashed green line) in two target range bins compared with the radar-measured noise (solid blue line) in those bins in the absence of RMP-simulated breathing for the entire duration of data collection (**a**), and for the interval between 10 s and 50 s (**b**).

A new pattern of the gantry noise that contains different speeds and pauses not previously seen by the ANN is tested to make sure that this ANN does not just stores a copy of the interference patterns, but can identify new patterns. Figure 7 shows the RB-ANN estimated interference signal compared with the radar-measured interference (in the target bins without RMP-simulated breathing) for the same targeted range bins of the previous input shown in Figure 6 (blue line in Figure 6a; bin 25). Shown also is interference estimation and comparison with reference noise for bin 21 (“b21”). The MSE on the estimated noise in the 11 target range bins is 9.4 × 10${}^{-8}$. This demonstrates the RB-ANN does not require retraining in order to estimate interference that varies from the training reference.

#### 3.2.3. Signal Enhancement Using Range Bin Weighted Averaging

**Figure 8:**
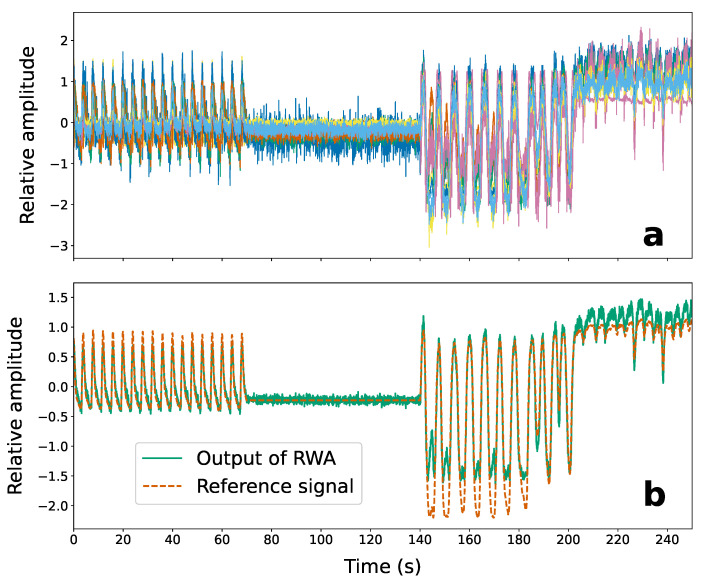
Recovered breathing signals from 11 target range bins before range-bin weighted averaging (RWA) (**a**), and after averaging (**b**) (solid green line). The reference breathing signal from the respirator motion phantom is also shown with the averaged signal (dashed orange line).

Figure 8 shows the effect of the range bin weighted averaging (Section 2.2.3) on the recovered target signals. The top of Figure 8a shows the weighted and polarity inverted breathing signals recovered from the 11 target range bins using the RB-ANN. Figure 8b shows the results of averaging (solid green line) compared with the reference breathing signal obtained from the RMP software (dashed orange line). The weights are obtained using the method presented by Zyweck and Bogner [37].

### 3.3. Combining ANN Noise Estimation, Range Bin Weighted Averaging and Filtering

**Figure 9:**
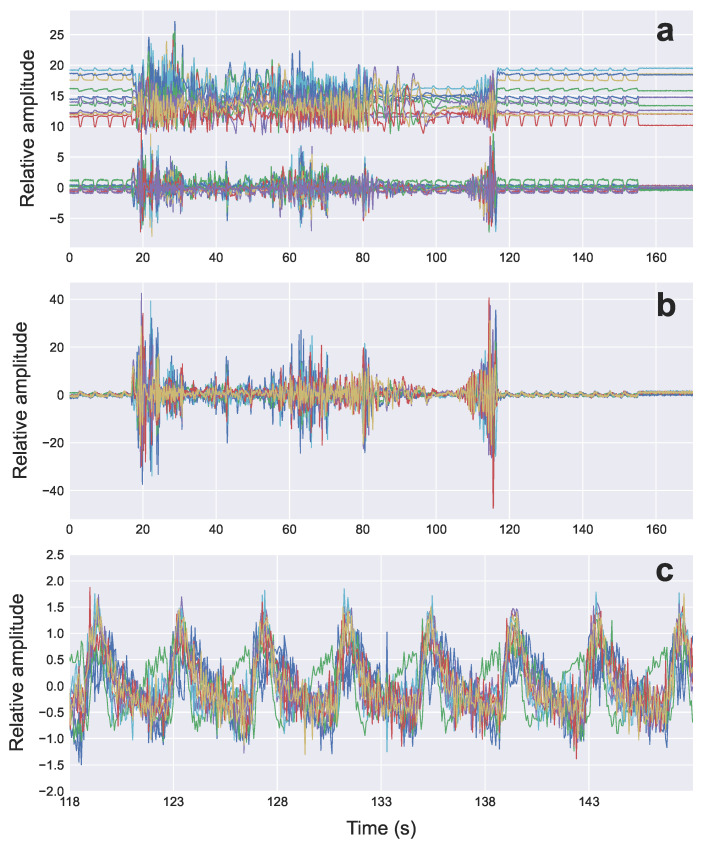
Raw RB-ANN filtered radar signals from 11 target range bins measured for a sinusoidal breathing pattern simulated by the RMP during VMAT treatment delivery (**a**). Each colored line represents the signal from one of the 11 range bins. Shown also are the weighted and polarity inverted signals for the entire duration of data collection (**b**) and for the interval between 110 s and 150 s (**c**).

**Figure 10:**
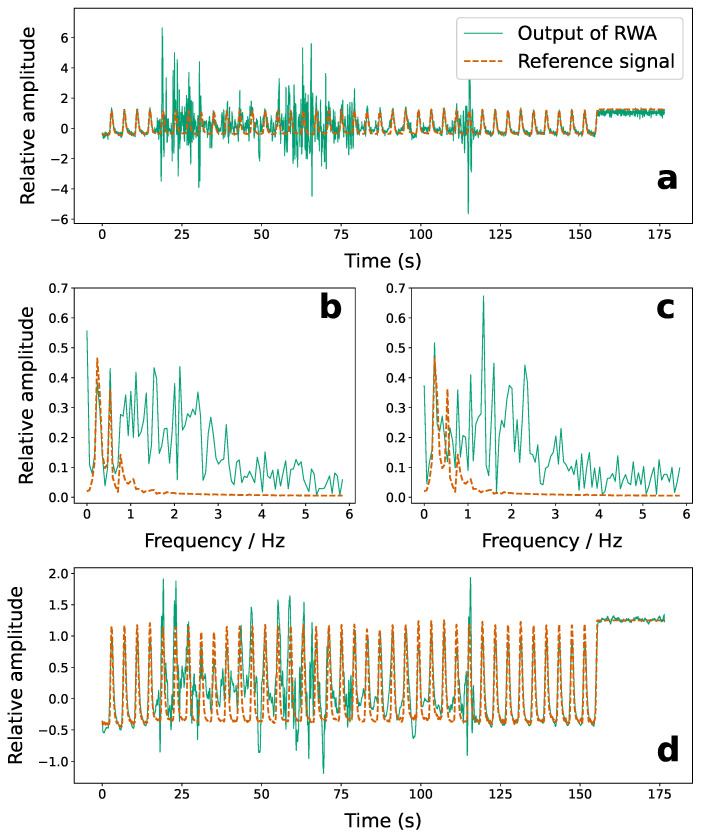
The range-bin weighted average (RBWA) radar signal from 11 target range bins measured for a sinusoidal breathing pattern simulated by the RMP during VMAT treatment delivery (**a**), along with the frequency domain of the signal collected from 18 to 36 s (**b**) and 57–75 s (**c**). The processed signal is shown in solid green while the reference signal from the RMP software is represented by the dashed orange line. Shown also is the weighted average signal after moving averaging filtering (**d**) overlapped with the reference signal from the RMP software.

In this section, the result of combining the RB-ANN noise estimation with range bin weighted averaging signal enhancement is demonstrated using a measurement of a sinusoidal breathing pattern programmed on the RMP during VMAT treatment delivery. The effect on the target range bin radar signals as it progresses through each signal processing step is shown in Figure 9 and Figure 10. Each colored line in Figure 9a represents the signal from one of the 11 range bins. Figure 9a shows the raw radar signal from the 11 target range bins before and after RB-ANN noise estimation. The weighted and polarity inverted signals are shown in Figure 9b,c. In this example, data was collected over a 180 s period, with RMP breathing motion on for the first 150 s, and treatment delivery occurring between 18 and 118 s.

The enhanced signal resulting from the range bin weighted averaging of the signals from Figure 9b are shown in Figure 10a (solid green line). Shown also is the breathing trace from the RMP software (dashed orange line). The frequency domain of the enhanced radar signal from 18 to 32 s and 57 to 75 s is shown in Figure 10b and Figure 10c, respectively, and highlights the remaining noise in those time intervals.

A moving average is used in applied to the signal shown in Figure 10a since it can be executed in real time and therefore be used to detect changes in breathing patterns with an acceptably small time delay. Figure 10d shows the result of using a moving average with a window size of 68 samples on the enhanced signal from Figure 10a (solid blue line). The reference signal from the RMP software is also shown (dashed red line).

### 3.4. Classification Performance

In this section, the two trained ANNs are applied to the entire data set (1.5 h of recorded treatment time) to test their performance on detecting the breathing signal with and without simultaneous linac operation. The resulting signals were passed through a moving-average (MA) filter using window of 17 samples. The filtered signals were then classified based on their power, and performance measured based on the power of a 68-sample moving window.

**Figure 11:**
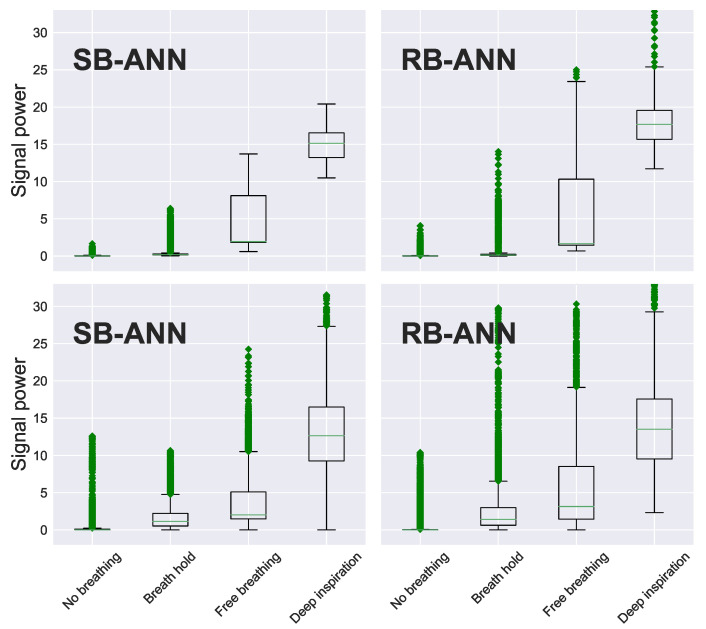
Box and whisker diagrams of radar-signal power for four categories of RMP-simulated breathing motion, with (gantry) and without (no-gantry) linac motion/operation, and after signal interference is removed: 1. No breathing (zero amplitude/signal power). 2. Breath hold (small amplitude/signal power), 3. Free (periodic) breathing, and 4. deep inspiration/exhalation (large amplitude/signal power). The boxes enclose the 2nd–3rd quartiles, while the whiskers outline the 2nd and 98th percentiles. The “+” symbols represent outliers.

Figure 11 shows box and whisker diagrams of the radar-measured signal power after filtering by an ANN for four categories of breathing, including 1. no breathing, 2. breath hold, 3. free (regular) breathing, and 4. deep inhalation/exhalation as outlined in Section 2.4. The box corresponds to the span between the first and third quartiles, with the middle line representing the median. The whiskers represent the 2nd and 98th percentiles, while the ‘+’s show outliers. Results are shown for signal measured both with and without linac motion/operation. There is a significant overlap in power level between the four classes; however, the relative radar-measured signal power increases, as expected, with breathing amplitude.

Sensitivity, specificity, and accuracy of the classifier were also quantified and summarized in Table 1. Sensitivity measures the ability of a classifier to include true positive in a group, and it is measured by dividing the number of true positive outcomes by the total number of true positives. The specificity measures the ability of a classifier to exclude true negatives from a group, and it is measured by dividing the number of true negative outcomes by the total number of true negatives. The overall accuracy is measured by dividing the number of true positives and true negative over the total number of measured power. Table 1 shows sensitivity and specificity calculated per each class in cases: 1. without and 2. with gantry interference. In addition, classification was performed after the signals were processed using both neural networks that we implemented: SB-ANN and RB-ANN. The results show that the sensitivity is significantly improved in case of strong gantry interference when RB-ANN noise estimation is used before the classification.

## 4. Discussion

The technologies that are currently used for respiration gating during radiation treatment are based on monitoring chest movement using contact-based sensors (breathing belts) or using contactless methods (cameras or lasers). Placing the breathing belt on a patient takes time and sometimes it is not well tolerated by the patient. Optical surface scanning uses a projector to generate light and cameras to detect the reflections from the motion of the chest surface. The main optical surface scanning technologies include laser scanners, time-of-flight systems, stereo vision systems and structured light systems [38]. Disadvantages of these systems are their complexity and price. There have been several works that investigate the range resolution of an UWB radar. It was claimed in [39] that movements of 2 mm of a phantom can be detected with Xethru X4M03 radar that we also used. Camera based systems can have better resolution (<1 mm) which is especially needed during breath hold intervals [38]. However, our results showed that our inexpensive system has high sensitivity in classifying all breathing types. One of the future tasks is to quantify the range resolution of the Xethru X4M03 radar as well as radars operating at higher bands (77 GHz) that have inherently better range resolution.

The use of a single, low-cost UWB radar device together with the breathing signal detection strategies presented here form a novel approach to real-time breathing motion detection, and represents an evolution over previous applications of radar technology in the radiotherapy setting. A robust implementation of radar for breathing motion monitoring has the added advantage of being a contactless technology and small form factor. The approaches to removing interference signals from the linac during dynamic treatment delivery, using the SB-ANN and RB-ANN, results in a level of accuracy, sensitivity, and specificity that makes the application of radar for breathing and general motion detection in this setting clinically viable. The ability of this system to classify breathing motion patterns is further improved during static gantry treatment delivery, such as is in the case of DIBH treatments of left-sided breast cancer patients using tangent POPs.

While radar-based motion monitoring system holds promise and has several distinct advantages, some improvements and considerations must be made regarding successful implementation. To begin with, the system is based on the assumption that the position of the radar is fixed and known in advance. In addition, we assume that the range bins in which breathing signal can be detected (about 10 range bins) as well as the range bins in which gantry motion can be detected are also known in advance. In our opinion, these are acceptable limitations since once the radar is fixed, the distance to the gantry and to the patient’s upper thorax are well localized. In addition, based on the nature of the signals (breathing is pseudoperiodic and gantry movement is observed as high-power noise) these distances can be estimated in semi-real time, which represents one direction of our future work. The next limitation is that the system needs to be run when there is no patient in order for the neural network to learn about the interference signal from the gantry. However, this step does not need to be repeated for each patient.

It is worth highlighting that two ANNs have been developed for the purpose of removing unwanted signal interference. SB-ANN cleans the interference signal in only one pre-selected range bin that corresponds to the chest, while RB-ANN cleans the interference signals from multiple range bins in which the breathing is detected. Since, the signals from multiple range bins are considered they need to be properly fused into one cleaned breathing signal. This is done by weighted averaging where the weights are also pre-determined based on the power of the signal obtained in each range bin. Again, this part can be improved in future work by learning the weights. In addition, the signals in some of the range bins are inverted (phase shifted by 180 degrees) because of the way the radar processes the signals internally. This is automatically recognized and corrected by our algorithm.

When the breathing signal is available, breathing rate estimation can easily be implemented by detecting the peak frequency in frequency domain or by counting the number of peaks that correspond to inhalations in time domain. In this way it is also possible to determine when the breathing rate is zero—i.e., breath hold or no patient present, which was one of the objectives in this paper. However, we did not implement this approach for detecting stop breathing events because breathing rate estimation requires the time window of several breathing periods in order to achieve the same accuracy. Since classification algorithm uses shorter time window for determining breath hold events (and other classes) we decided to implement simple classification algorithm to detect breath hold events.

The classification approach shows promise. Future work will include supervised learning techniques for performing classification. Significant loss in sensitivity was due to misclassification between no breathing (no patient) and the breath hold class. In practice, this will not represent a problem because the system will not need to be operational when there is no subject. Furthermore, there is a misclassification between regular breathing and hold breathing in case the subject is struggling to perform a breath hold, and therefore causing chest displacement. By including additional features, such as for example frequency context of the breathing signal, we believe that we will be able to address this problem. In previous work [21], 43 features were combined to distinguish between different human activities. Future reduction allowed us to achieve similar results with six features. Therefore, more work that is outside of the scope of this paper will be done on designing a proper classifier.

We consider extending the work by using novel compact multisensor device (radar, microphone arrays, cameras) that will allow for more accurate detection of motion and interference and will assist in improving the accuracy of localization of the patient’s chest.

## 5. Conclusions

Two methods for processing the signals from UWB radar measurements of breathing motion in the presence of a high-noise background have been implemented and evaluated. The methods were combined to form an algorithm for detecting breathing motion during radiotherapy using UWB radar. It has also been shown to suppress high noise power and recover simulated breathing signal in with acceptable accuracy. Further improvements are expected with adaptive filters, wavelet, singular-value decomposition and principal component analysis to guide the ANN training, while the output of these methods is used as an input to a simple classifier, future work will include developing classifier based on a larger number of features.

## Figures and Tables

**Table 1:** Summary of the sensitivity, specificity, and accuracy of the signal processing procedure consisting of ANN noise estimation, range bin weighted averaging and moving average filtering for four different breathing patterns with and without background noise from gantry motion during VMAT treatment delivery.

		No-Gantry Noise		Gantry Noise	
		Sensitivity	Specificity	Sensitivity	Specificity
SB-ANN	No breathing	0.86	0.97	0.86	0.86
	Hold breathing	0.73	0.89	0.47	0.87
	Regular breathing	0.90	0.96	0.66	0.87
	Deep inspiration	1	0.99	0.83	0.98
	Overall accuracy	0.85		0.72	
RB-ANN	No breathing	0.90	0.97	0.98	0.98
	Hold breathing	0.72	0.92	0.78	0.78
	Regular breathing	0.79	0.96	0.87	0.87
	Deep inspiration	1	0.99	0.97	0.97
	Overall accuracy	0.88		0.70	

## Data Availability

The code and the data presented in this study is made publicly available at https://codeocean.com/capsule/3291217/tree/v1, accessed on 13 December 2021.

## Abbreviations

The following abbreviations are used in this manuscript:

VMAT	Volumetric modulated arc therapy
DIBH	deep-inspiration breath hold
UWB	ultra-wideband
RMP	respiratory motion phantom
ANN	artificial neural network
SB-ANN	single-bin ANN
RB-ANN	range-bin ANN
RBWA	range-bin weighted averaging
SNR	Signal to Noise Ratio
MSE	Mean Square Error
MA	moving-average
